# Genetic evidence for causal association between migraine and dementia: a mendelian randomization study

**DOI:** 10.1186/s12920-024-01956-x

**Published:** 2024-07-05

**Authors:** Qiuyi Chen, Chengcheng Zhang, Shiyang Wu, Yiwei He, Yuhan Liu, Libin Zheng, Bin Li, Guiyou Liu, Lu Liu

**Affiliations:** 1 Beijing Key Laboratory of Acupuncture Neuromodulation, Department of Acupuncture and Moxibustion, Beijing Hospital of Traditional Chinese Medicine, Capital Medical University, Beijing, 100010 China; 2 Beijing Institute of Brain Disorders, Laboratory of Brain Disorders, Collaborative Innovation Center for Brain Disorders, Ministry of Science and Technology, Capital Medical University, Beijing, 100069 China; 3 State Key Laboratory of Networking and Switching Technology, Beijing University of Posts and Telecommunications, Beijing, 100876 China

**Keywords:** Migraine, Dementia, Alzheimer’s disease, Vascular dementia, Frontotemporal dementia, Dementia with Lewy bodies, Mendelian randomization

## Abstract

**Background:**

There is an association between migraine and dementia, however, their causal relationship remains unclear. This study employed bidirectional two-sample Mendelian randomization (MR) to investigate the potential causal relationship between migraine and dementia and its subtypes: Alzheimer’s disease (AD), vascular dementia (VaD), frontotemporal dementia (FTD), and dementia with Lewy bodies (DLB).

**Methods:**

Summary-level statistics data were obtained from publicly available genome-wide association studies (GWAS) for both migraine and five types of dementia. Single nucleotide polymorphisms (SNPs) associated with migraine and each dementia subtype were selected. MR analysis was conducted using inverse variance weighting (IVW) and weighted median (WM) methods. Sensitivity analyses included Cochran’s Q test, MR pleiotropy residual sum and outlier (MR-PRESSO) analysis, the intercept of MR-Egger, and leave-one-out analysis.

**Results:**

Migraine showed a significant causal relationship with AD and VaD, whereas no causal relationship was observed with all-cause dementia, FTD, or DLB. Migraine may be a potential risk factor for AD (odds ratio [OR]: 1.09; 95% confidence interval [CI]: 0.02–0.14; *P* = 0.007), while VaD may be a potential risk factor for migraine (OR: 1.04; 95% CI: 0.02–0.06; *P* = 7.760E-5). Sensitivity analyses demonstrated the robustness of our findings.

**Conclusion:**

Our study suggest that migraine may have potential causal relationships with AD and VaD. Migraine may be a risk factor for AD, and VaD may be a risk factor for migraine. Our study contributes to unraveling the comprehensive genetic associations between migraine and various types of dementia, and our findings will enhance the academic understanding of the comorbidity between migraine and dementia.

**Supplementary Information:**

The online version contains supplementary material available at 10.1186/s12920-024-01956-x.

## Introduction

Migraine is a highly prevalent neurological disorder characterized by severe headache, throbbing, and associated symptoms such as photophobia, phonophobia, nausea, and vomiting [1]. It affected approximately 14.4% of the world population and was considered the second largest contributor to the disability, owing to pain and recurrent attacks [2]. People who suffer from migraine is twice as likely as the general population to develop dementia, and migraine is linked to an increased risk of subsequent dementia [3, 4]. Although there may be an association between migraine and dementia, migraine pathophysiology remains poorly understood and requires further exploration. Dementia primarily includes Alzheimer’s disease (AD), vascular dementia (VaD), frontotemporal dementia (FTD), and Lewy body dementia (DLB). Several researches have reported an increased risk of all-cause dementia, AD, or VaD in patients with migraine compared to individuals without migraine [5–7]. However, little research has been conducted on the relationship between migraine and FTD or DLB.

Furthermore, previous research has demonstrated that individuals diagnosed with migraine exhibit a heightened susceptibility to dementia, with this correlation being predominantly observed among female patients [8]. Attention, memory, and language skills are impaired in patients with migraine because they develop abnormalities in the white matter at a faster rate than those without migraine. Damage to the white matter of the brain, where neuronal fibers gather, can lead to the above symptoms of dementia [9, 10]. In addition, depression is a common comorbidity between migraine and dementia, which suggests a central mechanism between migraine and dementia [11, 12]. Notably, these results indicate that migraine may be a risk factor for dementia, and identifying the risk factors for dementia may help in the early identification of individuals at risk and the development of prevention strategies. However, the results of these studies are not completely consistent, and research exploring the causal association between migraine and dementia is lacking.

Traditional observational studies’ conclusions are usually impacted by confounding factors and cannot establish causation. Additionally, randomized clinical trials may be limited by ethical considerations. Therefore, effective strategies must be developed to determine the causal relationships between exposure factors and response outcomes. Mendelian randomization (MR) was proposed in the 1990s, and with the emergence of large-scale genome-wide association studies (GWAS), MR has increasingly improved the research framework and methods [13, 14]. MR uses single nucleotide polymorphism (SNP) associations from GWAS to study and uncover the causal relationships between complex traits [15]. In our study, we used bidirectional two-sample MR analysis to investigate the genetic relationships between migraine risk and dementia, as well as performed a causal inference analysis.

## Materials and methods

### Study design

**Fig. 1:**
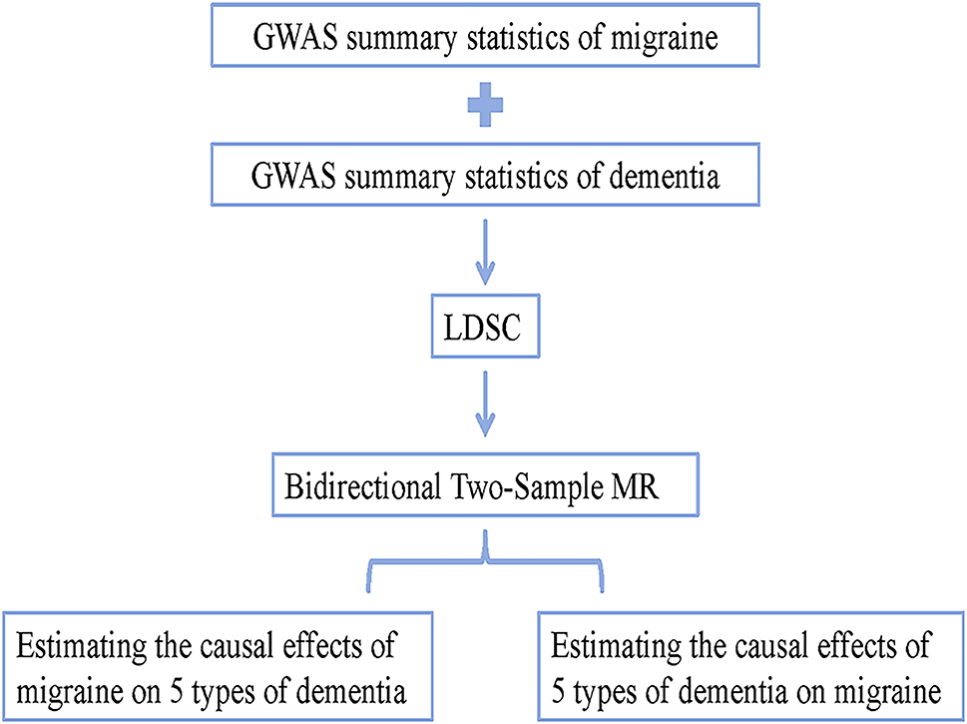
Overview of this bidirectional MR study design

This study was designed as a bidirectional two-sample MR research study, an overview of which is presented in Fig. 1. First, we used linkage disequilibrium score regression (LDSC) to evaluate the genetic links between migraine and the five types of dementia. Second, we conducted an MR analysis and a series of sensitivity analyses to assess the causal relationship between migraine and dementia. Finally, we employed reverse MR to determine whether dementia was linked with migraine.

**Fig. 2:**
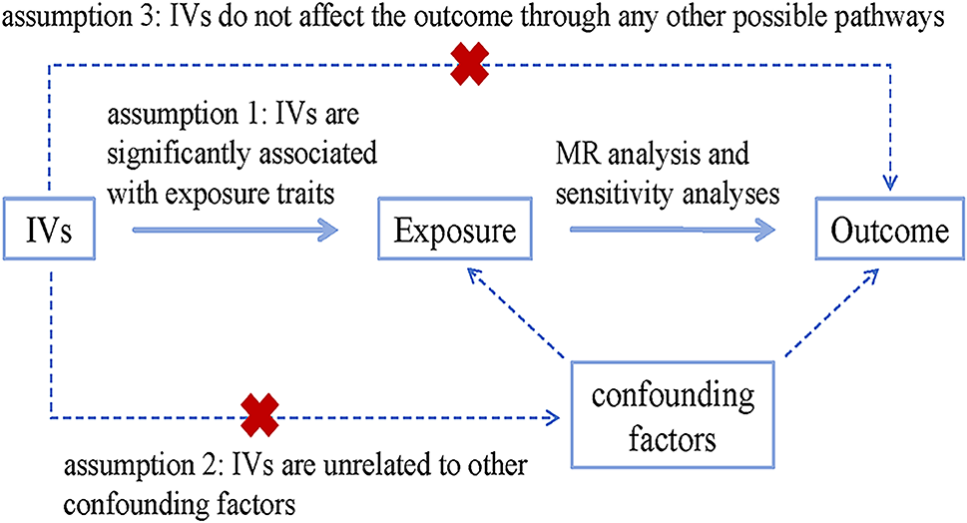
IVs should satisfy three core assumptions

To ensure that the causal relationships derived from the MR analysis are reliable, instrumental variables (IVs) should satisfy three core assumptions: (1) IVs are significantly associated with the exposure feature, (2) IVs are unrelated to other confounding factors, and (3) IVs do not affect the outcome through any other possible pathways (Fig. 2) [14, 16].

### Data sources

We acquired GWAS summary-level data for migraine from the 2022 International Headache Genetics Consortium (IHGC), which aggregates datasets from the UK Biobank, GeneRISK, HUNT, and the GWAS of the 2016 IHGC, for a total of 24 cohorts, 48,975 patients with migraine and 540,381 migraine-free individuals of European ancestry [17]. This 2022 IHGC migraine GWAS contained association results for 123 independent risk loci. To protect the privacy of participants in the 23andMe cohort, the migraine GWAS summary statistics applied in our analysis excluded individuals from this cohort (Table 1).

The summary-level GWAS dataset for AD was derived from a recent GWAS that included 788,989 individuals (111,326 patients and 677,663 controls) from Europe [18]. We acquired GWAS summary statistics for VaD from the FinnGen Consortium (Round 9, https://www.finngen.fi/en), comprising a total of 363,113 individuals of European descent (2,335 patients and 360,778 controls). The GWAS summary statistics for FTD were obtained from a study conducted by Ferrari et al. This study included a total of 12,932 individuals, consisting of 3,526 patients and 9,402 control individuals [19]. The DLB GWAS dataset was extracted from a study of 6,618 individuals (2,591 patients and 4,027 controls) [20]. Table 1 presents the essential characteristics of the dataset included in this study.

**Table 1:** Details of GWAS datasets included in MR analysis

Year	Trait	Population	Cases	Controls	Sample size	Web sources
2022	Migraine	European	48,975	540,381	589,356	10.1038/s41588-021-00990-0
2022	AD	European	111,326	677,663	788,989	10.1038/s41588-022-01024-z
2023	VaD	European	2,335	360,778	363,113	www.finngen.fi/en
2014	FTD	European	3,526	9,402	12,932	10.1016/S1474-4422(14)70065-1
2021	DLB	European	2,591	4,027	6,618	10.1038/s41588-021-00785-3
2023	Any Dementia	European	16,499	356,660	373,159	www.finngen.fi/en

*AD* Alzheimer’s disease, *VaD* vascular dementia, *FTD* frontotemporal dementia, *DLB* dementia with Lewy bodies

### Ethical approval

All summary-level datasets in our study were obtained from de-identified public data/studies. Ethical approval and informed consent were previously obtained from the ethics committee. Thus, the requirement for ethical approval was waived for this study.

### Instrumental variables selection

The key to the selection of IVs is to ensure the accuracy and robustness of the causal inferences. The following steps were performed: First, single nucleotide polymorphisms (SNPs) that were strongly linked with exposures were screened at genome-wide significance (*P* < 5 × 10^− 8^). For FTD and VaD, only a few or no SNPs reached the significance threshold (*P* < 5 × 10^− 8^), so we chose a more relaxed threshold (*P* < 1 × 10^− 5^) to select instrumental variables. This threshold was used in previous MR studies when a limited number of SNPs met the conventional threshold [21–23]. Second, we merged the IVs with the aforementioned significance and the outcome data to avoid losing too many IVs; therefore, we did not search for SNPs. The identified SNPs underwent clumping for LD using a stringent threshold of clumping r² < 0.001 within a 10,000 kb window. LD was estimated using European samples from the 1000 Genome Project as a reference [24]. The IVs are listed in Supplementary Table S1. Thirdly, in order to evaluate the presence of weak instrument bias, we utilized the F statistic, which was calculated according to previous study described methods, to assess the association of the retained SNPs [25]. SNPs with an F statistic < 10 were regarded as weak instruments and were subsequently excluded from the analysis [26]. The F-statistic is calculated using the following formula: F = R² × (*N* − 2)/(1 − R²); R² = 2 × EAF × (1 − EAF) × β², in which, R² refers to the cumulative explained variance of the selected IVs on migraine, EAF refers to the effect allele frequency, β refers to the estimated effect of SNPs, and N refers to the sample size of the GWAS [27]. Because of the lack of an FTD EAF, we did not calculate the R² and F statistics for the FTD. Finally, after harmonizing the exposure and outcome datasets and excluding palindromic SNPs, the remaining SNPs were utilized for the MR analysis.

### Statistical analysis

To assess the causal impact of migraine on dementia, we conducted the random-effects inverse variance-weighted (IVW) method in a two-sample MR analysis, which is extensively used in MR investigations and offers reliable causal effect estimation. We employed the weighted median method to further validate the results, and this method often exhibits larger positive causal effects, especially as the number of invalid instrumental variables increased. Combining these two analysis methods can enhance the reliability of the conclusions [28]. Moreover, we performed extensive sensitivity analyses to analyze potential model assumptions violations in the MR study and improve the robustness of the MR results. Cochran’s Q test was used to analyze the heterogeneity of individual causal effects. Furthermore, MR pleiotropy residual sum and outlier (MR-PRESSO) analysis was conducted to detect outlier instrumental variables [29], and horizontal pleiotropy was examined using the MR-PRESSO global test. In order to minimize the influence of horizontal pleiotropy, outlier IVs found by the MR-PRESSO outlier test were deleted step by step. The MR-PRESSO distortion test was used to analyze whether there were significant differences in the results before and after excluding outliers, and the MR-Egger intercept was used to determine pleiotropy [30]. Finally, we performed a leave-one-out analysis to explore the impact of excluding each selected individual SNP on the overall results. Moreover, we conducted a reverse MR analysis to test the potential for reverse causation (i.e., we assessed the effects of dementia on migraine). Statistical significance was set at *P* < 0.05 each analysis, and statistical power was calculated using an online tool at https://shiny.cnsgenomics.com/mRnd [31]. Owing to the lack of an FTD EAF, we did not calculate the statistical power for FTD when it was used as an exposure risk factor. The statistical analysis carried out in R 4.3.1 using the ‘TwoSampleMR’ package (version 0.5.7) [32].

## Results

When migraine was considered as an exposure factor under the more stringent threshold (*P* < 5 × 10^− 8^ and clumped at LD r² = 0.001), 35 SNPs were selected for any dementia, AD, VaD, and DLB, and 33 SNPs for FTD, after harmonization. When the five types of dementia were considered as exposure factors, under the condition of *P* < 5 × 10^− 8^ and clumped at LD r² = 0.001, 14 SNPs were selected for any dementia, 51 SNPs for AD, and five SNPs for DLB. Additionally, under the condition of *P* < 1 × 10^− 5^, we chose 23 SNPs for VaD and 10 SNPs for FTD. The calculated F-statistics of these SNPs were 136.50-51700.56, suggesting that strong instrumental variables were used (Table 2). In the forward MR analysis of migraine and AD, the statistical power was > 80%. When dementia and VaD were considered as risk factors, a statistical power of > 80% was observed in the reverse MR analysis (Table 2). Detailed information on the SNPs associated with migraine and the five types of dementia is provided in Supplementary Table S1.

**Fig. 3:**
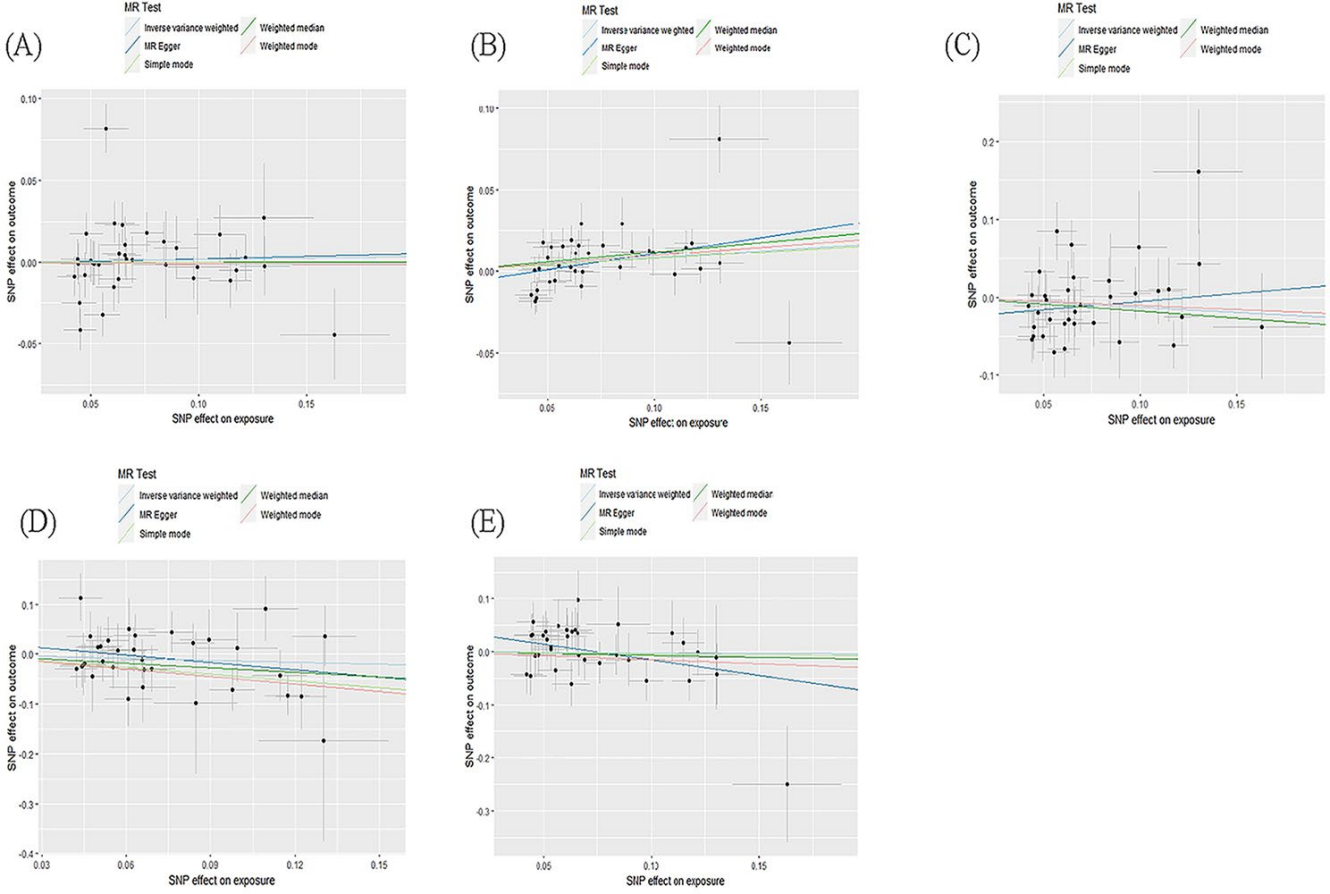
Scatter plots of the causal impact of migraine on dementia. The scatter plots show the causal effects of migraine on any dementia **(A)**, Alzheimer’s disease **(B)**, vascular dementia **(C)**, frontotemporal dementia **(D)**, dementia with lewy bodies **(E)**, when migraine is the risk factor. Three lines reveal the estimated effect sizes by MR methods (IVW, MR-Egger and weighted median)

**Fig. 4:**
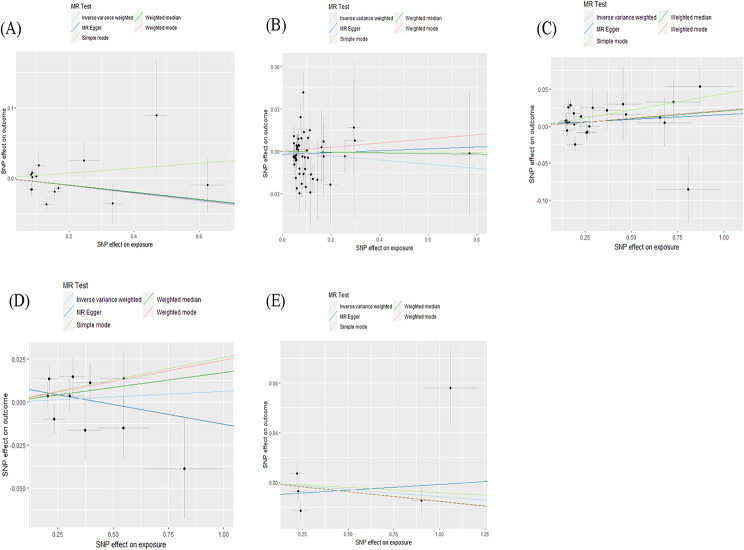
Scatter plots of the causal impact of dementia on migraine. The scatter plots show the causal effects of any dementia **(A)**, Alzheimer’s disease **(B)**, vascular dementia **(C)**, frontotemporal dementia **(D)**, dementia with lewy bodies **(E)** on migraine, when five types of dementia are the risk factors. Three lines reveal the estimated effect sizes by MR methods (IVW, MR-Egger and weighted median)

**Fig. 5:**
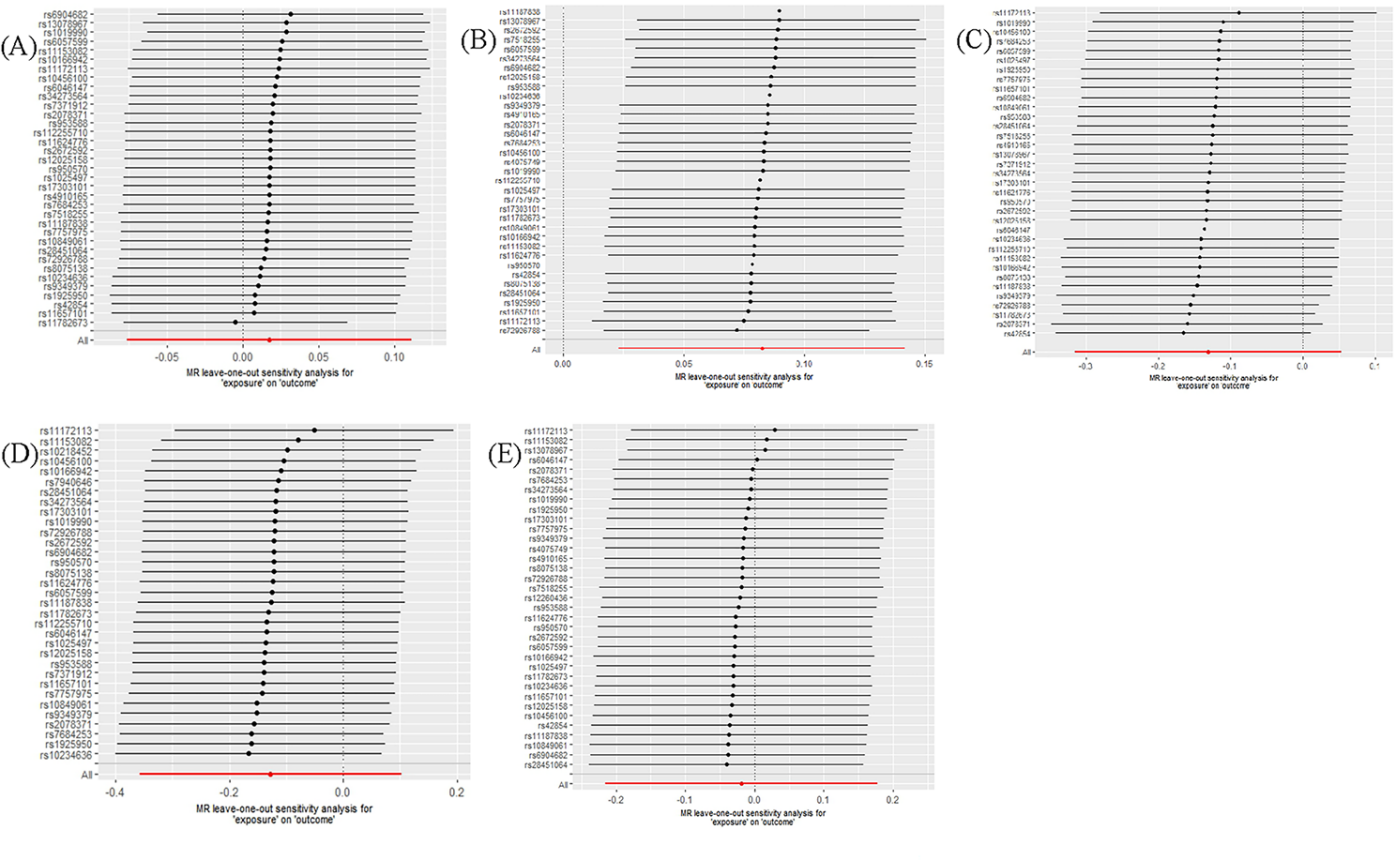
Leave-one-out plots of the causal impact of migraine on dementia. Leave-one-out plots show the association between any dementia **(A)**, Alzheimer’s disease **(B)**, vascular dementia **(C)**, frontotemporal dementia **(D)**, dementia with lewy bodies **(E)** and migraine when migraine is the risk factor, using IVs that excluded 1 SNP at a time from the overall instrumental variable or using IVW as an MR method to exclude all SNPs. Bars show effect size and 95% confidence interval

**Fig. 6:**
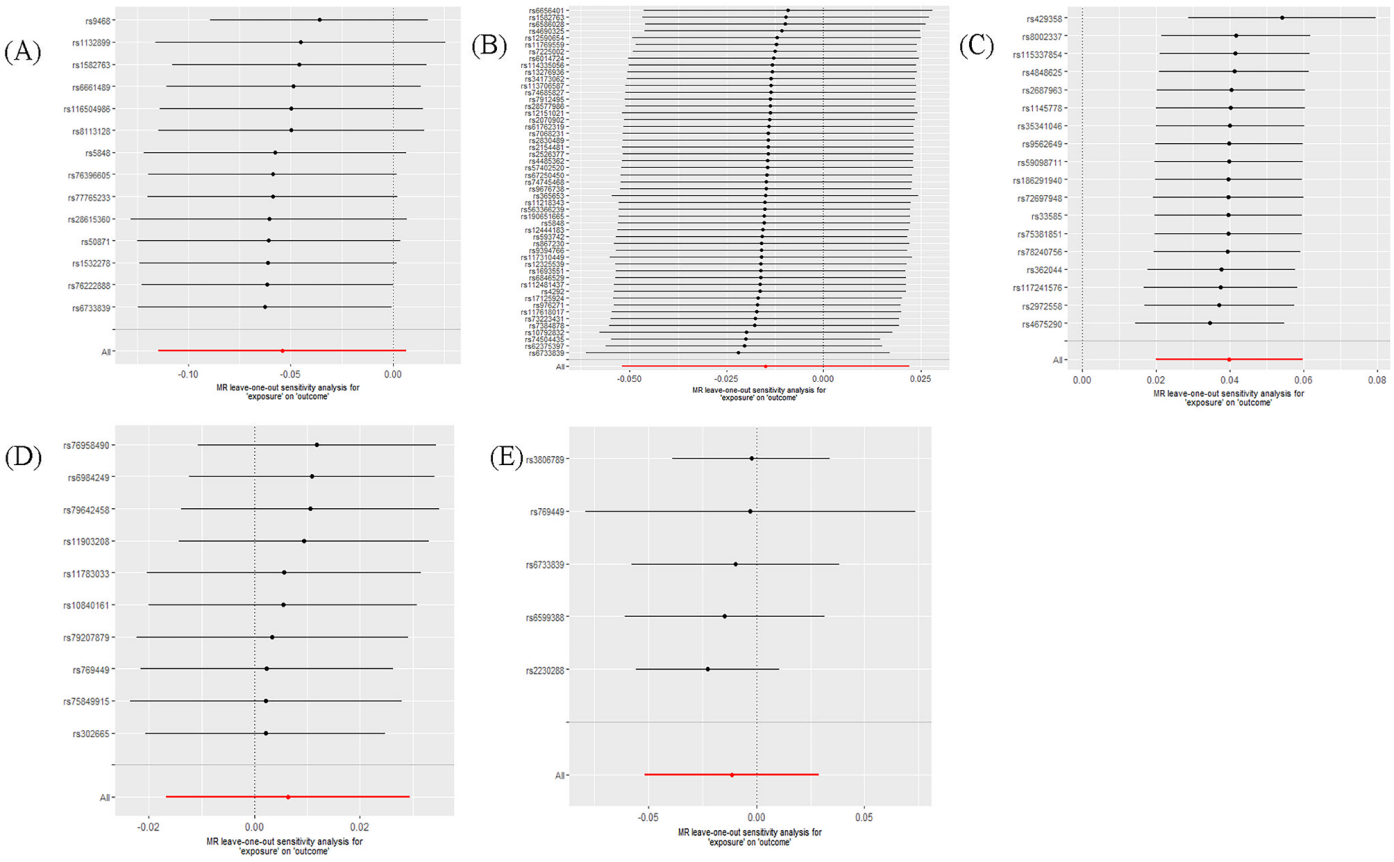
Leave-one-out plots of the causal impact of dementia on migraine. Leave-one-out plots show the association between any dementia **(A)**, Alzheimer’s disease **(B)**, vascular dementia **(C)**, frontotemporal dementia **(D)**, dementia with lewy bodies **(E)** and migraine when 5 types of dementia are the risk factors, using IVs that excluded 1 SNP at a time from the overall instrumental variable or using IVW as an MR method to exclude all SNPS. Bars show effect size and 95% confidence interval

The main results of the bidirectional MR analysis are presented in Table 3. The scatter and leave-one-out plots for each analysis are shown in Figs. 3, 4, 5 and 6.

### Forward MR analysis

For any dementia, using the IVW method, we did not identify any potential causal relationship between migraine and any dementia (odds ratio [OR]: 1.02; 95% confidence interval [CI]: -0.08–0.11; *P* = 0.711), which is consistent with the results obtained from the weighted median method (OR: 1.00; 95% CI: -0.09–0.09; *P* = 0.994). In the sensitivity analysis, the MR analysis revealed some heterogeneity (Cochran’s Q, *P* < 0.001). The Global test using the MR PRESSO method detected a certain level of horizontal pleiotropy (*P* < 0.001), and the subsequent outlier test identified two SNPs. However, the results of the distortion test showed no difference before and after correction (*P* = 0.39), and the intercept from the MR-Egger test was not significant (*P* = 0.910, Table 3). Furthermore, the leave-one-out plot indicated that no SNP had a significant impact on the outcome (Fig. 5).

For AD, consistent results from both the IVW (OR: 1.09; 95% CI: 0.02–0.14; *P* = 0.007) and weighted median methods (OR: 1.13; 95% CI: 0.05–0.18; *P* = 3.000E-04) revealed a positive genetic correlation with migraine. The sensitivity analysis revealed heterogeneity (Cochran’s Q, *P* = 0.001) and horizontal pleiotropy (MR PRESSO Global test, *P* = 0.002), and MR PRESSO outlier testing identified one SNP. However, the results of the distortion test (*P* = 0.614) and the intercept of the MR-Egger’s test (*P* = 0.178) were not statistically significant (Table 3). Furthermore, the leave-one-out plot indicated the robustness of the findings (Fig. 5).

For VaD, this MR study did not find any potential risk relationship between VaD and migraine using the IVW (OR: 0.88; 95% CI: -0.32–0.05; *P* = 0.163) and weighted median methods (OR: 0.84; 95% CI: -0.42–0.07; *P* = 0.158). Moreover, the sensitivity analysis did not reveal any heterogeneity (Cochran’s Q *P* = 0.100), horizontal pleiotropy (MR PRESSO Global test *P* = 0.098), or pleiotropy (MR Egger intercept test *P* = 0.098; Table 3). Furthermore, the leave-one-out plot visually demonstrates the stability of the results (Fig. 5).

For FTD, the IVW (OR: 0.88; 95% CI: -0.36–0.10; *P* = 0.277) and weighted median methods (OR: 0.74; 95% CI: -0.65–0.05; *P* = 0.089) did not reveal any potential causal effect between FTD and migraine. Additionally, no evidence of heterogeneity (Cochran’s Q, *P* = 0.628), horizontal pleiotropy (MR PRESSO Global test, *P* = 0.617), or pleiotropy (MR Egger intercept test, *P* = 0.276) was observed (Table 3). Furthermore, the stability of the results was demonstrated by the leave-one-out plot (Fig. 5).

For DLB, using the IVW (OR: 0.98; 95% CI: -0.22–0.18; *P* = 0.850) and weighted median methods (OR: 0.93; 95% CI: -0.36–0.22; *P* = 0.621), we did not identify any potential causal effect. The sensitivity analysis showed a slightly significant result in the pleiotropy test (MR-Egger intercept test, *P* = 0.047); however, the results of the heterogeneity test (Cochran’s Q, *P* = 0.604) and MR PRESSO Global test for horizontal pleiotropy (*P* = 0.615) were not statistically significant (Table 3). Furthermore, the leave-one-out plot supported the credibility of the results (Fig. 5).

### Reverse MR analysis

For the reverse MR analysis, we used the IVW method to investigate whether the five types of dementia are potential causal risk factors for migraine. Notably, we did not find any potential causal effects of dementia, AD, FTD, or DLB on migraine. In the reverse MR analysis for VaD, the IVW method did not show statistical significance (OR: 1.02; 95% CI: 0.00–0.04; *P* = 0.060). However, the sensitivity analysis revealed heterogeneity (Cochran’s Q *P* = 0.034) and horizontal pleiotropy (MR PRESSO Global test *P* = 0.047), and the leave-one-out plot indicated a potential bias from SNPs influencing the results. After excluding the five SNPs with significant effects from the leave-one-out analyses, we conducted a reanalysis with the filtered SNPs and identified a significant causal effect of VaD on migraine (OR: 1.04; 95% CI: 0.02–0.06; *P* = 7.760E-05). The results did not exhibit significant heterogeneity (Cochran’s Q, *P* = 0.638), pleiotropy (MR Egger intercept test, *P* = 0.122), or horizontal pleiotropy (MR PRESSO Global test, *P* = 0.576; Table 3), revealing the robustness and credibility of the MR analysis results (Fig. 6).

**Table 2:** The characteristics of each group in MR analysis

Exposure	Outcome	Number of SNPs	Significance threshold	The sum of cumulative F-values	Statistical power
Migraine	Any Dementia	35	*P* < 5E − 08	40420.95	0.09
Migraine	AD	35	*P* < 5E − 08	40404.30	1.00
Migraine	VaD	35	*P* < 5E − 08	40420.95	0.34
Migraine	FTD	33	*P* < 5E − 08	38639.63	0.42
Migraine	DLB	35	*P* < 5E − 08	40202.89	0.05
Any Dementia	Migraine	14	*P* < 5E − 08	28856.95	0.90
AD	Migraine	51	*P* < 5E − 08	113298.85	0.19
VaD	Migraine	23	*P* < 1E − 05	135030.93	0.82
FTD	Migraine	10	*P* < 1E − 05	NA	NA
DLB	Migraine	5	*P* < 5E − 08	1768.96	0.23

*SNP* Single-nucleotide polymorphism, *AD* Alzheimer’s disease, *VaD* vascular dementia, *FTD* frontotemporal dementia, *DLB* dementia with Lewy bodies

**Table 3:** The main results of the bidirectional MR analysis

Exposure	Outcome	Method	OR	95% CI	*P* value	Cochran’s Q test	MR PRESSO Global test	MR PRESSO distortion test	MR Egger
Migraine	Any Dementia	IVW	1.02	-0.08–0.11	0.711	*P* < 0.001	*P* < 0.001	*P* = 0.39	*P* = 0.910
		weighted median	1.00	-0.09–0.09	0.994				
Migraine	AD	IVW	1.09	0.02–0.14	0.007	*P* = 0.001	*P* = 0.002	*P* = 0.614	*P* = 0.178
		weighted median	1.13	0.05–0.18	3.000E-04				
Migraine	VaD	IVW	0.88	-0.32–0.05	0.163	*P* = 0.100	*P* = 0.098	NA	*P* = 0.098
		weighted median	0.84	-0.42–0.07	0.158				
Migraine	FTD	IVW	0.88	-0.36–0.10	0.277	*P* = 0.628	*P* = 0.617	NA	*P* = 0.276
		weighted median	0.74	-0.65–0.05	0.089				
Migraine	DLB	IVW	0.98	-0.22–0.18	0.850	*P* = 0.604	*P* = 0.615	NA	*P* = 0.047
		weighted median	0.93	-0.36–0.22	0.621				
Any Dementia	Migraine	IVW	0.95	-0.11–0.01	0.080	*P* = 0.028	*P* = 0.042	*P* = 0.214	*P* = 0.953
		weighted median	0.95	-0.11–0.01	0.123				
AD	Migraine	IVW	0.99	-0.05–0.02	0.427	*P* = 0.027	*P* = 0.032	NA	*P* = 0.526
		weighted median	1.00	-0.05–0.05	0.924				
VaD	Migraine	IVW	1.04	0.02–0.06	7.760E-05	*P* = 0.638	*P* = 0.576	NA	*P* = 0.122
		weighted median	1.03	0.00–0.05	6.612E-02				
FTD	Migraine	IVW	1.01	-0.02–0.03	0.587	*P* = 0.301	*P* = 0.325	NA	*P* = 0.351
		weighted median	1.02	-0.01–0.05	0.244				
DLB	Migraine	IVW	0.99	-0.05–0.03	0.577	*P* = 0.002	*P* = 0.049	*P* = 0.337	*P* = 0.535
		weighted median	0.98	-0.04–0.00	0.193				

*AD* Alzheimer’s disease, *VaD* vascular dementia, *FTD* frontotemporal dementia, *DLB* dementia with Lewy bodies. *IVW* inverse variance weighted, *WM* weighted median, *OR* odds ratio, *95% CI* 95% confidence interval, *MR PRESSO* Mendelian randomization pleiotropy residual sum and outlier. The significance threshold of 0.05 was used for *P*-values in each analysis

## Discussion

To the best of our knowledge, this is the first bidirectional two-sample MR analysis conducted to assess the causal relationship between migraine and dementia, including its subtypes. In our MR study, we primarily investigated the potential causal relationships between migraine and the five types of dementia. Our study contributes to unraveling the genetic associations between migraine and various types of dementia, and our findings will facilitate the advancement of clinical medicine and enhance the academic understanding of the comorbidity between migraine and dementia.

We found a positive association between genetically predicted migraine and AD, and this causal relationship remained significant when using the weighted median method. Although some bias risks were identified in heterogeneity and horizontal pleiotropy detection, pleiotropy was not significant, and the MR-PRESSO distortion test showed no statistical difference in the results before and after outlier correction. Notably, the leave-one-out plot analysis also revealed the robustness of the results. In the context of the reverse MR analysis, after removing five SNPs with substantial bias effects based on the leave-one-out plot, VaD ultimately exhibited a significant causal effect on migraine.

Several scholars have investigated the association between migraine and dementia. For instance, in a meta-analysis, researchers have found that migraine may be associated with an increased risk of all-cause dementia, AD, and VaD [33]. However, our results do not genetically support migraine as a risk factor for all-cause dementia or VaD. Additionally, a previous study conducted a MR analysis to appraise the potential influences of 18 neurodegenerative and neuropsychiatric disorders on AD. They found that four disorders, including migraine, bipolar disorder, schizophrenia, and Parkinson’s disease, are causally associated with an increased risk for AD [34]. These findings are consistent with our study results. Although our study suggests that VaD may be an important risk factor for migraine, the association between migraine and VaD remains controversial. Some studies have indicated a causal relationship between migraine and dementia, particularly VaD [35]. However, another study suggested a significant association between migraine and all-cause dementia and AD, but not VaD [36]. The heterogeneity among the different research conclusions may be attributed to differences in study designs, such as the specific diagnostic subtypes of patients with migraine, geographical variations, follow-up duration, and different outcome measures (all-cause dementia, AD, or VaD) included in previous cohort studies. In addition, cohort studies often face challenges in terms of obtaining adequate sample sizes and sufficient statistical power. In this study, we used publicly available large-scale databases to provide robust statistical power. Additional research is warranted to elucidate the association between migraine and dementia, validating the findings of our study.

Our study suggests that migraine may have potential causal relationships with AD and VaD. Specifically, migraine may be a risk factor for AD, and VaD may be a risk factor for migraine. Given these associations, several potential future directions for clinical practice emerge. First, routine cognitive impairment screenings for migraine patients could facilitate early detection of cognitive decline, enabling timely interventions to potentially slow dementia progression. Second, considering the complex relationship between migraine and various cerebrovascular conditions, early management of vascular risk factors is crucial. This could involve regular monitoring and control of blood pressure, lipid levels, and other vascular health indicators in migraine patients, thereby reducing the incidence of vascular events and associated dementia risk. Third, developing individualized treatment plans by tailoring strategies based on the specific medical history and risk factors of migraine patients is essential. For instance, migraine patients with a family history of hypertension or cardiovascular diseases might benefit from more aggressive preventive measures. Lastly, encouraging close collaboration between neurologists, cardiovascular and cerebrovascular specialists can provide a more holistic assessment and management of these patients’ health risks, improving overall outcomes.

This study has several limitations. First, the bidirectional two-sample MR analysis offers advantages over traditional observational studies, as it can mitigate confounding factors and distinguish between the directions of causal relationships. Second, comprehensive sensitivity analyses were conducted to ensure the robustness and credibility of the results. Additionally, we categorized dementia into five types, to provide richer information and more reliable results.

Nevertheless, our study design and results had some limitations. First, our data sources were limited to European populations, which limits the generalizability of our findings to other populations. Second, owing to the lack of explicit differentiation between chronic and episodic migraine in the available data, we were unable to perform additional MR analyses. Moreover, because the GWAS data were aggregated, subgroup analyses were not feasible. Third, some heterogeneity and pleiotropy were observed during the analysis, which may introduce biases that need to be interpreted in conjunction with the sensitivity analysis results. Fourth, although we made efforts to select the largest available datasets to date, some combinations of exposures and outcomes may have low statistical power owing to the small sample size, potentially leading to false-negative results. Thus, future large-scale GWAS are needed to explore the association between migraine and dementia. Fifth, regarding FTD and VaD as exposure variables, the significance threshold for SNP selection was relatively low. Although the F-statistic suggests a low likelihood of weak instrument bias, false-positive variations and the resulting biases cannot be completely avoided, and caution is advised when interpreting the significant causal effect of VaD on migraine. For VaD and FTD, further MR analyses should be conducted when a sufficient number of SNPs are obtained by screening using conventional thresholds. Sixth, there may be sample overlap between dementia, VaD, and migraine in some data sources, which could lead to a winner’s curse and weak instrument bias. Further MR analyses should be conducted with larger sample sizes and different data sources from other GWAS studies, as they become available.

## Conclusion

Our finding suggests that migraine may have a potential causal relationship with AD and VaD. On one hand, migraine may be a risk factor for AD, while on the other hand, VaD may be a risk factor for migraine. Our research contributes to a better understanding of the causal relationship among migraine, dementia, and its subtypes from a genetic perspective, filling a gap in the academic field and enhancing the academic understanding of the comorbidity between migraine and dementia. Further exploration of the shared biological mechanisms underlying migraine and dementia is required. Updated MR studies will be necessary to validate our findings when more extensive GWAS summary data are available.

### Electronic supplementary material

Below is the link to the electronic supplementary material.

Supplementary Material 1

## Data Availability

The data used in this study are publicly available. The complete migraine dataset is available for download from the 2022 International Headache Genetics Consortium (IHGC) website: http://www.headachegenetics.org. The dementia GWAS summary results are publicly available for download from the FinnGen Consortium (https://www.finngen.fi/en) and three articles (10.1038/s41588-022-01024-z; 10.1016/s1474-4422(14)70065-1; 10.1038/s41588-021-00785-3).

## Abbreviations

MR: Mendelian randomization
AD: Alzheimer’s disease
VaD: Vascular dementia
FTD: Frontotemporal dementia
DLB: Dementia with Lewy bodies
GWAS: Genome-wide association studies
SNPs: Single nucleotide polymorphisms
IVW: Inverse variance weighting
WM: Inverse variance weighting
MR-PRESSO: Mendelian randomization pleiotropy residual sum and outlier
LDSC: Linkage disequilibrium score regression
IVs: Instrumental variables
IHGC: International Headache Genetics Consortium
OR: Odds ratio
95% CI: 95% Confidence interval
